# IGF2BP3 functions as a potential oncogene and is a crucial target of miR-34a in gastric carcinogenesis

**DOI:** 10.1186/s12943-017-0647-2

**Published:** 2017-04-11

**Authors:** Yuhang Zhou, Tingting Huang, Ho Lam Siu, Chi Chun Wong, Yujuan Dong, Feng Wu, Bin Zhang, William K. K. Wu, Alfred S. L. Cheng, Jun Yu, Ka Fai To, Wei Kang

**Affiliations:** 1 Department of Anatomical and Cellular Pathology, State Key Laboratory in Oncology in South China, Prince of Wales Hospital, The Chinese University of Hong Kong, Hong Kong, SAR People’s Republic of China; 2 Partner State Key Laboratory of Digestive Disease, Institute of Digestive Disease, The Chinese University of Hong Kong, Hong Kong, SAR People’s Republic of China; 3 Sir Y.K. Pao Cancer Center, Li Ka Shing Institute of Health Science, The Chinese University of Hong Kong, Hong Kong, SAR People’s Republic of China; 4 Shenzhen Research Institute, The Chinese University of Hong Kong, Shenzhen, People’s Republic of China; 5 Department of Gastroenterology, The Affiliated Drum Tower Hospital of Nanjing University, Medical School, Nanjing, People’s Republic of China; 6 Department of Anaesthesia and Intensive Care, The Chinese University of Hong Kong, Hong Kong, SAR People’s Republic of China; 7 School of Biomedical Sciences, The Chinese University of Hong Kong, Hong Kong, SAR People’s Republic of China; 8 Department of Medicine and Therapeutics, The Chinese University of Hong Kong, Hong Kong, SAR People’s Republic of China

**Keywords:** Gastric cancer, IGF2BP3, miR-34a

## Abstract

**Background:**

Gastric cancer (GC) is one of the frequent causes of cancer-related death in eastern Asian population. IGF2BP2 lists in the top rank up-regulated genes in GC, but its functional role is unclear.

**Method:**

The expression of IGF2BP3 in GC cell lines and primary samples was examined by qRT-PCR and Western blot. The biological role of IGF2BP3 was revealed by a series of functional in vitro studies. Its regulation by microRNAs (miRNAs) was predicted by TargetScan and confirmed by luciferase assays and rescue experiments.

**Results:**

IGF2BP3 ranked the No.1 of the up-regulated genes by expression microarray analysis in GC cell lines. The expression level of IGF2BP3 was observed in GC tissues comparing with non-tumorous gastric epitheliums. The up-regulated IGF2BP3 expression was associated with poor disease specific survival. IGF2BP3 knockdown significantly inhibited cell proliferation and invasion. Apart from copy number gain, IGF2BP3 has been confirmed to be negatively regulated by tumor-suppressive miRNA, namely miR-34a. The expression of miR-34a showed negative correlation with IGF2BP3 mRNA expression in primary GC samples and more importantly, re-overexpression of IGF2BP3 rescued the inhibitory effect of miR-34a.

**Conclusion:**

We compressively revealed the oncogenic role of IGF2BP3 in gastric tumorigenesis and confirmed its activation is partly due to the silence of miR-34a. Our findings identified useful prognostic biomarker and provided clinical translational potential.

**Electronic supplementary material:**

The online version of this article (doi:10.1186/s12943-017-0647-2) contains supplementary material, which is available to authorized users.

## Background

Gastric cancer (GC) is one of the most prevalent malignancies worldwide. Accordingly, its incidence ranks the 4th in men and 5th in women while it causes the 3rd cancer-related death for men and 5th for women [1]. Although the incidence appears to decrease in recent years, the mortality still remains high, which may due to the delayed diagnosis and the lack of effective treatment. Its occurrence and development often arise from the interaction between internal genetic heterogeneity and multiple external risk factors, such as *Helicobacter Pylori* infection and high-salt diet [2]. For decades, this severe disease has attracted public attention. In 1965, Lauren P identified two types of GC, diffuse and intestinal, based on different histological features [3], which has been widely used in clinical pathology since then. Until 2010, another histological classification was suggested by World Health Organization (WHO): tubular, papillary, mucinous and poorly cohesive (including signet ring cell carcinoma), plus uncommon histologic variants [4]. In 2014, The Cancer Genome Atlas (TCGA) proposed a novel characterization which was built upon the molecular mechanisms, and this classification leads to a brand new insight and an in-depth understanding of GC [5].

Many new emerging technologies are employed in GC research including expression microarray. By screening the putative dysregulated genes achieved by expression microarray analysis in nine GC cell lines, we found IGF2BP3 (Insulin-like growth factor-2 mRNA-binding protein 3) listing in the No.1 rank of the up-regulated genes.

IGF2BP3, also known as IMP3, belongs to a conserved IGF2 mRNA-binding protein family. IGF2BP3 was first identified due to its high abundance in pancreatic carcinoma [6]. After its initial identification, IGF2BP3 has soon been explicated to be a mainly over-expressed member among the family in various tumor types, such as squamous cell carcinoma [7], lung cancer [8], melanoma [9], colon cancer [10], liver cancer [11]. And the aberrant upregulation implicated its potential oncogenic role in tumorigenesis. Furthermore, accumulating evidences demonstrated that IGF2BP3 represented a promising biomarker in different cancers, such as colon cancer [12] and GC [13]. However, knowledge of its function and regulation in GC is still quite limited.

High expression of IGF2BP3 mRNA but with low copy number gain rate, suggested that post-transcriptional regulation might play an important role for the IGF2BP3 upregulation in GC. By bioinformatic analysis, we found IGF2BP3 might be regulated by miR-34a (www.microrna.org), which was listed in the relative top rank. microRNAs (miRNAs) has been thought to be new regulators of gene expression through binding to the 3' untranslated regions (UTRs) of the targeted mRNAs [14], and then degrade or translationally inhibit those targeted mRNAs. Deviant expressions of miRNAs were detected in various human malignancies [15], and the aberrant expression is always correlated with oncogenesis [16, 17].

Thus in current study, we will firstly investigate the basic expression and functional role of IGF2BP3 in GC. Secondly, we will comprehensively reveal the expression regulation of IGF2BP3 by miR-34a in GC and detect their expression correlation in primary samples. Finally, the clinical correlation and survival-prediction significance of these potential biomarkers will be revealed. Collectively, we aim to deeply explore the molecular mechanism of up-regulated IGF2BP3 in gastric tumorigenesis and offer a translational potential for clinical intervention of GC.

## Methods

### GC cell lines and primary gastric tumor samples

Human GC cell lines (MKN1, MKN7, MKN28, MKN45, SNU1, SNU16, AGS, KatoIII, NCI-N87, MGC-803, SGC-7901, TMK-1) and immortalized gastric epithelial cells (GES-1 and HFE-145) were described previously [18]. Cells were cultured at 37 °C in humidified air atmosphere containing 5% CO2 in RPMI 1640 (GIBCO, Grand Island, NY) medium supplemented with 10% fetal bovine serum (GIBCO). A cohort of 247 formalin-fixed paraffin embedded tissues of GCs diagnosed between 1999 and 2006 in the Prince of Wales Hospital, Hong Kong was retrieved. Ethical approval was obtained from the Joint Chinese University of Hong Kong-New Territories East Cluster Clinical Research Ethics Committee.

### RNA extraction and quantitative real-time polymerase chain reaction (qRT-PCR)

Cultured cells were harvested for extracting total RNA with TRIzol reagent (Invitrogen, Carlsbad, CA). cDNA synthesis was performed with a High-Capacity cDNA Reverse Transcription Kits (Applied Biosystems, Carlsbad, CA). The variations of mRNA expression of related genes were quantified by qRT-PCR and primers were listed as following: IGF2BP3 (sense: AGT TGT TGT CCC TCG TGA CC; anti-sense: GTC CAC TTT GCA GAG CCT TC); B2M (sense: ACT CTC TCT TTC TGG CCT GG; anti-sense: ATG TCG GAT GGA TGA AAC CC). The protocol of qRT-PCR was described in a previous study [19]. For microRNA expression detection, miR-34a expression in GC was detected by Taqman miRNA assays, and they were used to quantify the levels of mature miR-34a (Assay ID: #4427975, Life Technologies). The relative expression level of miR-34a was normalized by RNU6B expression (Assay ID: #001093). 7500 Fast Real-Time System (Applied Biosystems) was used for the qPCR reaction. And the reaction system was incubated at 95 °C for 30 s, followed by 40 cycles of 95 °C for 8 s and 60 °C for 30 s.

### Protein extraction and western blot analysis

The protein extraction and western blot analysis protocol were described in our previous study [20]. The primary antibodies detected IGF2BP3 (#07-104) was from Merck Millipore. Other primary antibodies were from Cell Signaling (Danvers, MA) including p21 (#2946), p27 (#2552), p-Rb (Ser807/811) (#9308), cleaved-PARP (Asp214) (#9541), Cyclin D3 (#2936), CDK4 (# 12790), CDK6 (#3136) and GAPDH (#2118). The secondary antibodies were anti-Mouse IgG-HRP (Dako, 00049039, 1:30000) and anti-Rabbit IgG-HRP (Dako, 00028856, 1:10000).

### Immunohistochemistry

Immunohistochemistry was to conduct tissue microarray within a 4 μm-thick section of each clinical sample using Ventana NexES automated Stainer (Ventana Corporation). All sections were performed microwaving in EDTA antigen retrieval buffer after de-waxing in xylene and graded ethanol. The IGF2BP3 primary antibody (1:100, 07–104) was from Merck Millipore. The cytoplasmic expression of IGF2BP3 was evaluated according to the proportion of tumor cells with intensity of cytoplasmic staining [20].

### miRNA and siRNA transfection for functional assays

The miRNA precursor miR-34a (PM11030) and scramble control (AM17110) were commercially available from Life Technologies. siIGF2BP3-1 (SI03230759) and siIGF2BP3-2 (SI04234167) were obtained from Qiagen (Valencia, CA). Lipofectamine 2000 Transfection Reagent (Invitrogen) was used for all transfection assays. The cell proliferation experiments, colony formation assays in monolayer, cell invasion assays, flow cytometry analysis for cell cycle distribution have been described in our previous work [21]. The experiments were repeated in triplicate to obtain standard deviations.

### Luciferase activity assays

The putative miR-34a binding site in IGF2BP3 3'UTR of was subcloned into pMIR-REPORT Vector (Ambion). The oligonucleotides that encompass the miR-34a recognition site and the oligonucleotides which contain the mutated binding site were listed in Additional file 1: Table S1. Prior to digestion and subcloning, oligonucleotides were annealed in 30 mmol/L HEPES buffer that contains 100 nmol/L potassium acetate and 2 mmol/L magnesium acetate [22]. The firefly luciferase constructs were co-transfected with Renilla luciferase vector control into MGC-803 cells. Dual luciferase reporter assays (Promega, Madison, WI) were performed 24 h after transfection.

### Treatment of cell lines with 5-Aza and TSA

AGS, MKN1, NCI-N87 and MGC-803 were treated with demethyltransferase inhibitor (5-Aza, Sigma, St Louis, MO) and histone-deacetylase inhibitor trichostatin A (TSA, Sigma) [23]. Cells were incubated with 10 μM 5-Aza for 72 h in 5-Aza group, while in TSA group, cells were treated with 100 nM TSA for 24 h. As for the combination treatment group, cells were treated with 5-Aza for 96 h and 100 nM TSA was added into the culture medium in the last 24 h. Equal amount of vehicle DMSO (Sigma) was used in negative control groups.

### In vivo tumorigenicity model

MGC-803 cells (10^7^ cells suspended in 0.1 ml PBS) were transfected with miR-34a or scramble control then were injected subcutaneously into the left and right dorsal flank of 4-week old Balb/c nude mice respectively. Diameters of tumors were measured and documented each 5 days with a total of 25 days. Tumor volume (mm^3^) was accessed by measuring the longest and shortest diameter of the tumor and calculating as follows: volume = (shortest diameter) ^2^ × (longest diameter) × 0.5. All animal handling and experimental procedures were approved by Department of Health in Hong Kong.

### Rescue experiments

For the rescue experiments, AGS and MKN28 cells were first transfected with miR-34a precursor or negative control respectively. After 24 h incubation, IGF2BP3 expression plasmid (#19879, Addgene) together with empty vector (pcDNA3.1, Life Technologies) were transfected to the cells using FuGENE HD Transfection Reagent (Roche, Nutley, NJ). After another 24 h, cells were harvested for functional study (MTT proliferation, monolayer colony formation, and cell invasion assays). The in vivo rescue experiments were performed using MGC-803 cells [24].

### Statistical analysis

The Student *T* test was used to compare the differences in functional differences between siIGF2BP3 and siScramble control transfected cells. It is also used to compare the biological behavior between miR-34a transfected cells and scramble miRNA transfectant counterparts. Nonparametric Pearson Chi-Square test was used to evaluate the correlation between IGF2BP3 expression and selected clinicopathologic parameters. Kaplan-Meier method was used to estimate the survival rate for each parameter and the equivalences of the survival curve were examined by log-rank statistics. For those parameters were found statistically significant in the univariate survival analysis (*P* < 0.05), the Cox proportional hazards model was employed to further evaluate them for multivariate survival analysis. All statistical analysis was performed by SPSS software (version 22.0; SPSS Inc). A two-tailed *P*-value of less than 0.05 was considered statistically significant and the *P*-value less than 0.001 was considered highly significant.

## Results

### IGF2BP3 is highly expressed in GC

**Fig. 1:**
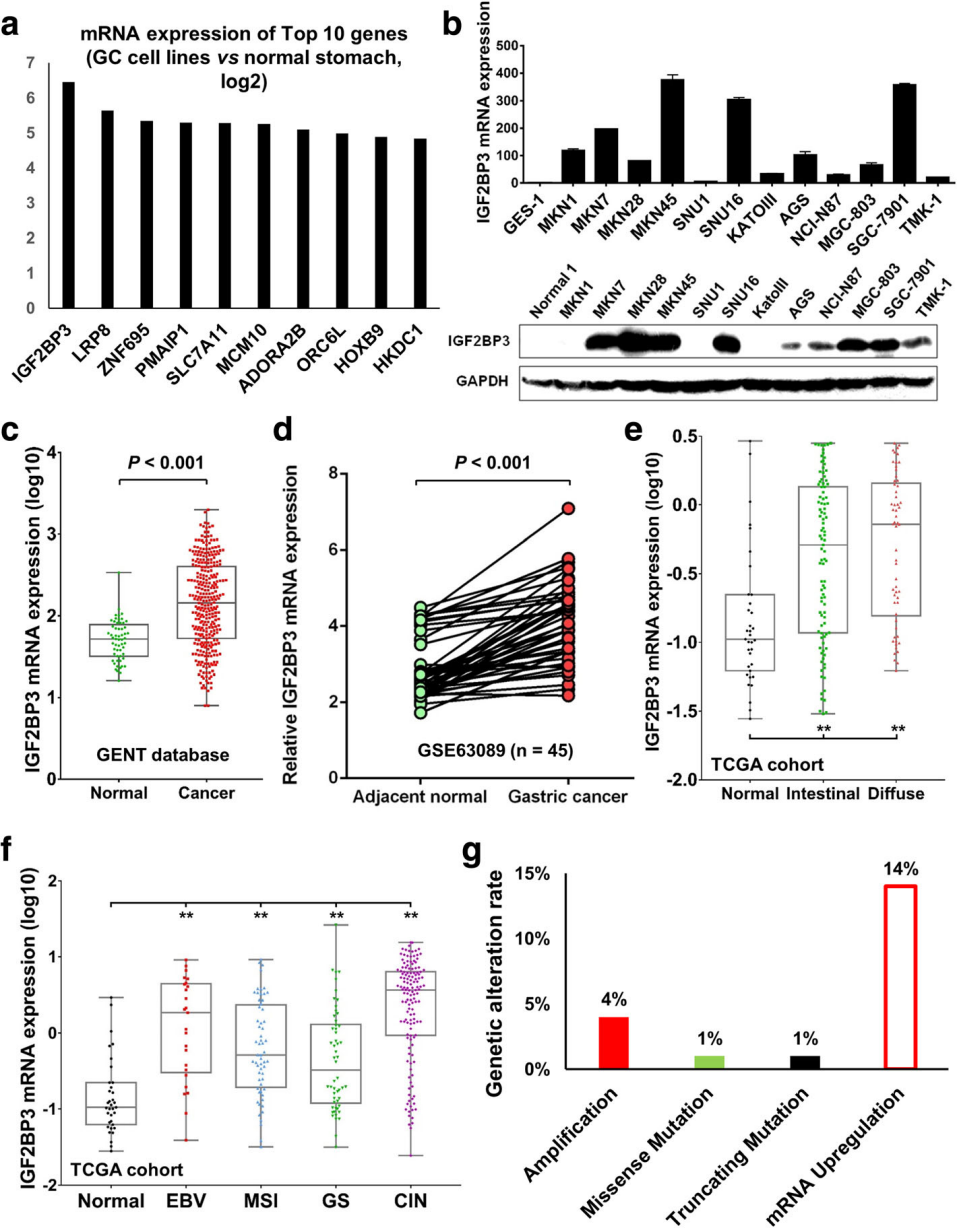
IGF2BP3 is up-regulated in GC. **a** Top ten most up-regulated genes from gene expression microarray analysis in nine GC cell lines. IGF2BP3 ranked the top one among the list. **b** The mRNA and protein expression of IGF2BP3 in 12 GC cell lines compared with immortalized gastric epithelium cell line GES-1. **c** IGF2BP3 was highly expressed in 311 GC samples compared with normal gastric tissues (from http://medical-genome.kribb.re.kr/GENT/, *P* < 0.001). **d** Increased expression of IGF2BP3 in primary gastric tumors compared with adjacent non-tumorous tissues (NCBI/GEO/GSE63089, *P* < 0.001). **e** mRNA expression of IGF2BP3 in intestinal and diffuse type GC (Lauren classification) (TCGA cohort, *n* = 193; **, *P* < 0.001) **f** IGF2BP3 mRNA expression in four molecular subtypes of GC (TCGA cohort, *n* = 313; **, *P* < 0.001). **g** Proportion of genetic alterations of IGF2BP3 (from http://www.cbioportal.org/; *n* = 258)

By gene expression microarray analysis in nine GC cell lines (Additional file 2: Table S2), IGF2BP3 was found to be the most up-regulated gene in the top ten list (Fig. 1a). Consistently, both mRNA and protein expression of IGF2BP3 were up-regulated in most of the GC cell lines comparing with immortalized gastric epithelium cell line (GES-1) (Fig. 1b). In primary samples, reports from GENT dataset manifested that IGF2BP3 showed a dramatically overexpression in 351 GC tissues when compared with the normal gastric tissues (*P* < 0.01, Fig. 1c) [25]. In addition, according to a published dataset NCBI/GEO/GSE63089 [26], IGF2BP3 was demonstrated to be highly expressed in GC samples compared with adjacent non-tumorous tissues (*n* = 45, *P* < 0.001, Fig. 1d). Moreover, based on TCGA cohort, IGF2BP3 showed significantly up-regulated in both intestinal- and diffuse- types of GC when compared with normal control (*P* < 0.001, Fig. 1e). In particular, when it came to the molecular characterization proposed by TCGA [5], high level of IGF2BP3 was observed frequently in the subtype of chromosomal instability (CIN) and EBV positive GC, while its level was relative lower in genomically stable (GS) subtype (Fig. 1f). For deep investigation of the regulation mechanisms of IGF2BP3 in GC, the genomic alteration of IGF2BP3 in TCGA cohort was analyzed by cBioPortal (www.cbioportal.org/) [27, 28], including copy number changes, somatic mutation and mRNA upregulation. It was found that 18% cases (47/258) have at least one alteration of IGF2BP3. And the rate of mRNA upregulation counted for 14% cases (Fig. 1g). Although the copy number gain showed positive correction with IGFBP3 mRNA expression (*P* < 0.05, Additional file 3: Figure S1), its mRNA upregulated in all GC samples can not merely be explained by copy number change.

### Overexpression of IGF2BP3 correlates with poor survival in GC

**Fig. 2:**
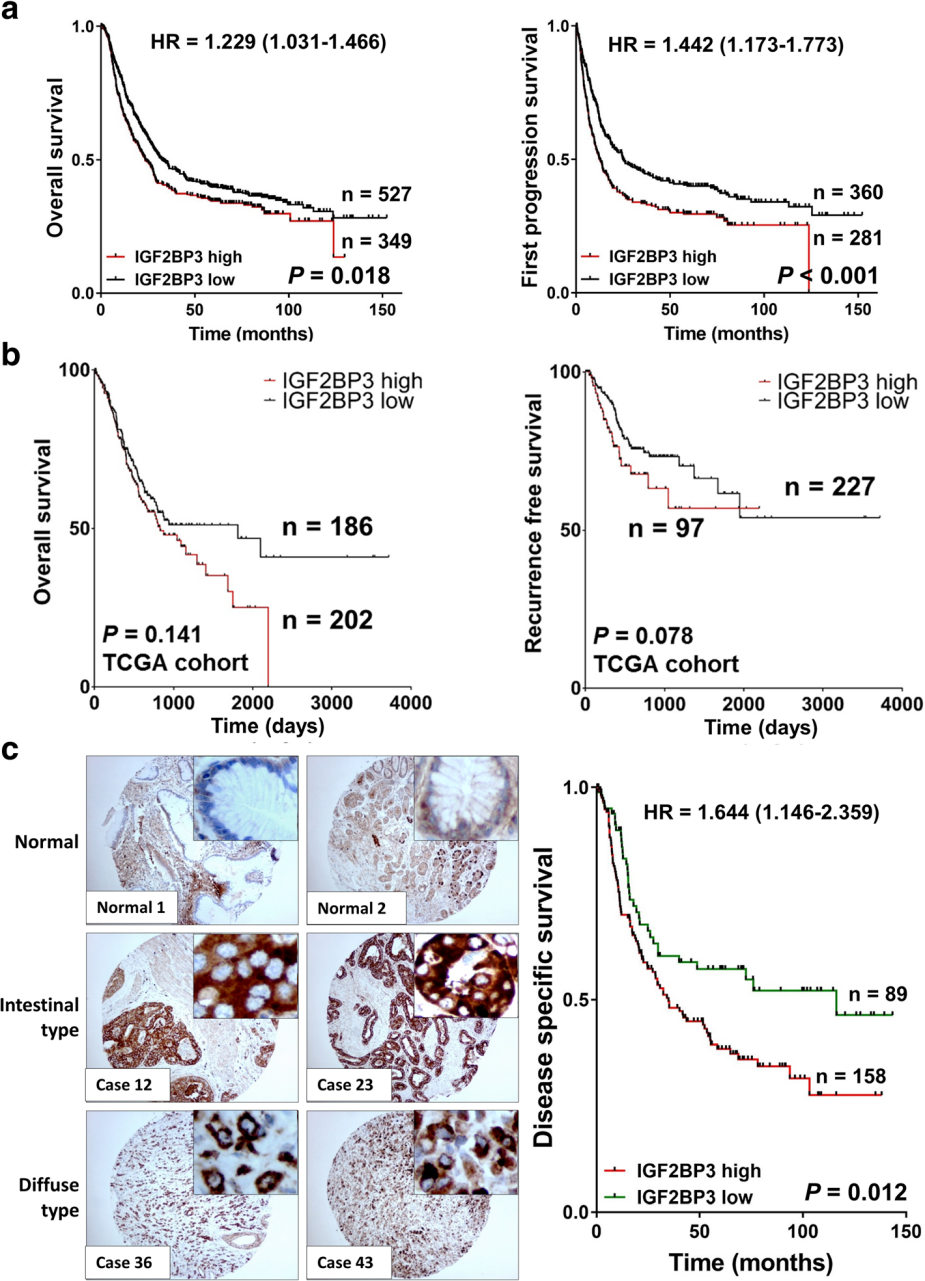
Overexpression of IGF2BP3 predicts poor prognosis in GC. **a** Over-expressed IGF2BP3 was related to worse overall outcome and significantly associated with first progression survival in primary GC samples from Kaplan Meier plotter. **b** In TCGA cohort, high IGF2BP3 expression showed a non-significance trend to predict unfavorable overall and recurrence free survival. **c** *Left panel*, representative immunohistochemistry images of IGF2BP3 in normal gastric epithelium tissues, intestinal-, diffuse type GC samples (original magnification × 100, insertion × 400). IGF2BP3 expression was mainly localized in the cytoplasm. Right panel, Kaplan-Meier plots of disease specific survival according to IGF2BP3 expression status. IGF2BP3 accumulation in cytoplasm (moderate/strong staining) was associated with poor disease specific survival in patients with GC (*P* = 0.012)

From Kaplan Meier plotter (www.kmplot.com), overexpression of IGF2BP3 was correlated with poor survival of both overall and first progression GC cases (*P* = 0.018, overall survival; *P* < 0.001, first progression survival; Fig. 2a and Additional file 4: Table S3) [29]. In TCGA cohort, although the *P*-value was not significant, there was still a trend that aberrant richness of IGF2BP3 was associated with unfavorable outcome (*P* = 0.141, overall survival; *P* = 0.078, recurrence free survival; Fig. 2b). Subsequently, we conducted immunohistochemistry to elucidate the protein expression of IGF2BP3 in GC tissue microarray. In normal gastric epitheliums, there was a negative expression of IGF2BP3. Significantly, a strong cytoplasmic staining was observed in both intestinal- and disuse-types of GC cells (left panel, Fig. 2c), and this strong staining predicted a poor disease specific survival if we classified the samples as two subgroups (negative/week and moderate/strong subgroups, *P* = 0.012, right panel, Fig. 2c). The correlations between IGF2BP3 and clinicopathologic parameters in 247 GC samples were summarized in Additional file 5: Table S4. Specifically, IGF2BP3 upregulation was only correlated with N stage (*P* = 0.025). In univariate analysis, age, diffuse type, high grade, advanced stage, lymph node metastasis and overexpression of IGF2BP3 had a statistical association with poor survival. However, by multivariate Cox regression analysis, only age and advanced stage were two independent factors (Additional file 6: Table S5).

### Silence of IGF2BP3 exerts tumor-suppressive effects both in vitro and in vivo

**Fig. 3:**
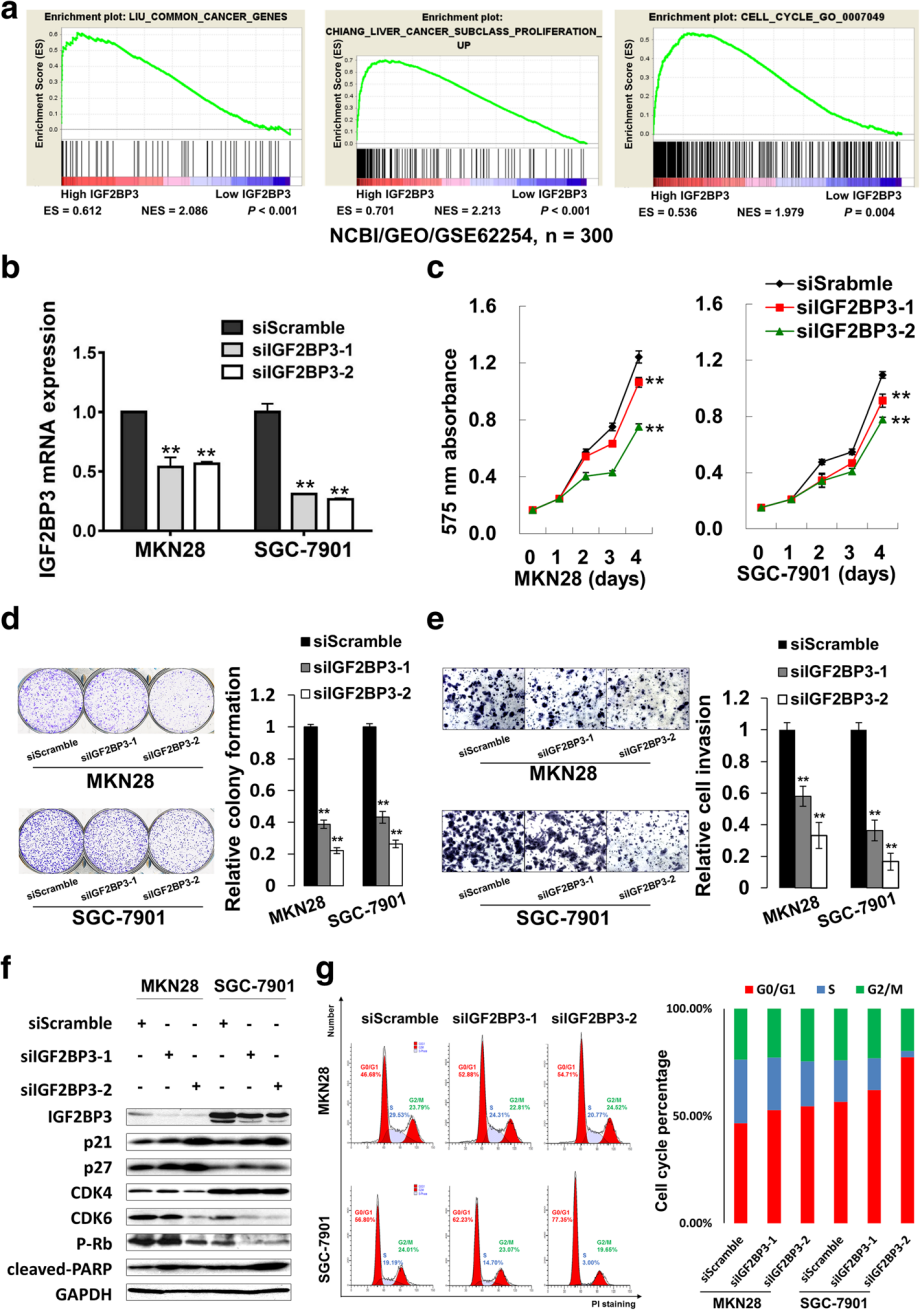
Silence of IGF2BP3 exerts anti-oncogenic role in GC. **a** Enrichment plots of gene expression signatures for common cancer genes (LIU_COMMON_CANCER_GENES) (*P* < 0.001), cell proliferation (CHIANG_LIVER_CANCER_SUBCLASS_PROLIFERATION_UP) (*P* < 0.001) and cell cycle progression (CELL_CYCLE_GO_0007049) (*P* = 0.004) according to IGF2BP3 mRNA expression in a published cohort (NCBI/GEO/GSE62254, *n* = 300). The *barcode plot* indexed the position of the genes in each gene set. *Red* and *blue* colors indicated *high* and *low level* of IGF2BP3. ES, enrichment score; NES, normalized enrichment score. **b** mRNA expression of IGF2BP3 after siRNA-mediated knockdown in MKN28 and SGC-7901 cells (**, *P* < 0.001). **c** Knocking down IGF2BP3 reduced cell growth in a 4-day MTT proliferation assay in MKN28 and SGC-7901 cells (**, *P* < 0.001). **d** Monolayer colony formation of GC cells was suppressed by siIGF2BP3 (**, *P* < 0.001). **e** IGF2BP3 knockdown inhibited cell invasive ability (**, *P* < 0.001). **f** Expression of related cell cycle regulators and apoptotic markers by Western blot analysis. CDK4, CDK6 and p-Rb were down-regulated, while p21 and p27 showed uniformly elevated expression in IGF2BP3-depleted cells. **g** Flow cytometry analysis of IGF2BP3 knockdown transfectants together with scramble siRNA transfectants as control. Two independent experiments were performed and the representative one was shown in the *bar chart*

From the results of GSEA (Gene Set Enrichment Analysis) in a published GC cohort NCBI/GEO/GSE62254 (*n* = 300) [30], IGF2BP3 upregulation was found to have a positive correlation with expression of a common cancer gene set which was defined by a group of Singaporean researchers (*P* < 0.001) [31]. Furthermore, IGF2BP3 was also significantly correlated with cell growth (*P* < 0.001) [32] and cell cycle progression (*P* = 0.004, Fig. 3a) [33, 34]. Since IGF2BP3 was expressed abundantly in MKN28 and SGC-7901 cell lines, we conducted siRNA-mediated knockdown experiments to reveal its function in these two cell lines. After treatment of two siIGF2BP3s, both mRNA and protein expressions of IGF2BP3 were declined in cells (Fig. 3b). Silencing IGF2BP3 slowed down the rate of cell growth, as indicated in a 4-day MTT assays (*P* < 0.001, Fig. 3c). Monolayer colony formation ability was inhibited after IGF2BP3 knockdown compared with scramble control (*P* < 0.001, Fig. 3d). Besides, cell invasive ability was markedly suppressed by siIGF2BP3 transfection (*P* < 0.001, Fig. 3e). As proliferation-inhibition phenotypes were observed in siIGF2BP3 groups, the associated cell cycle regulators and apoptosis markers were analyzed by Western blot. The phosphorylated Rb (p-Rb), CDK4, CDK6 expression were decreased but p21 and p27 were uniformly up-regulated in IGF2BP3 knockdown cells, supporting the G0/G1-phase cell cycle arrest. Together, knockdown of IGF2BP3 did not induce obvious late apoptosis in MKN28 and SGC-7901 cell (Fig. 3f). Then the transfectants for cell-cycle parameters were analyzed by flow cytometry to validate the Western blot results. As shown in Fig. 3g, MKN28 cells with siIGF2BP3 knockdown groups contained a higher percentage of G0/G1 phase cells (52.9 and 54.7% respectively) compared with siScramble control counterparts (46.7%). The cell population of G0/G1 phase in IGF2BP3-knockdown SGC-7901 cells were increased from siScramble control (56.8%) to 62.2 and 77.4% respectively (Fig. 3g).

### IGF2BP3 is a direct target of miR-34a in GC

**Fig. 4:**
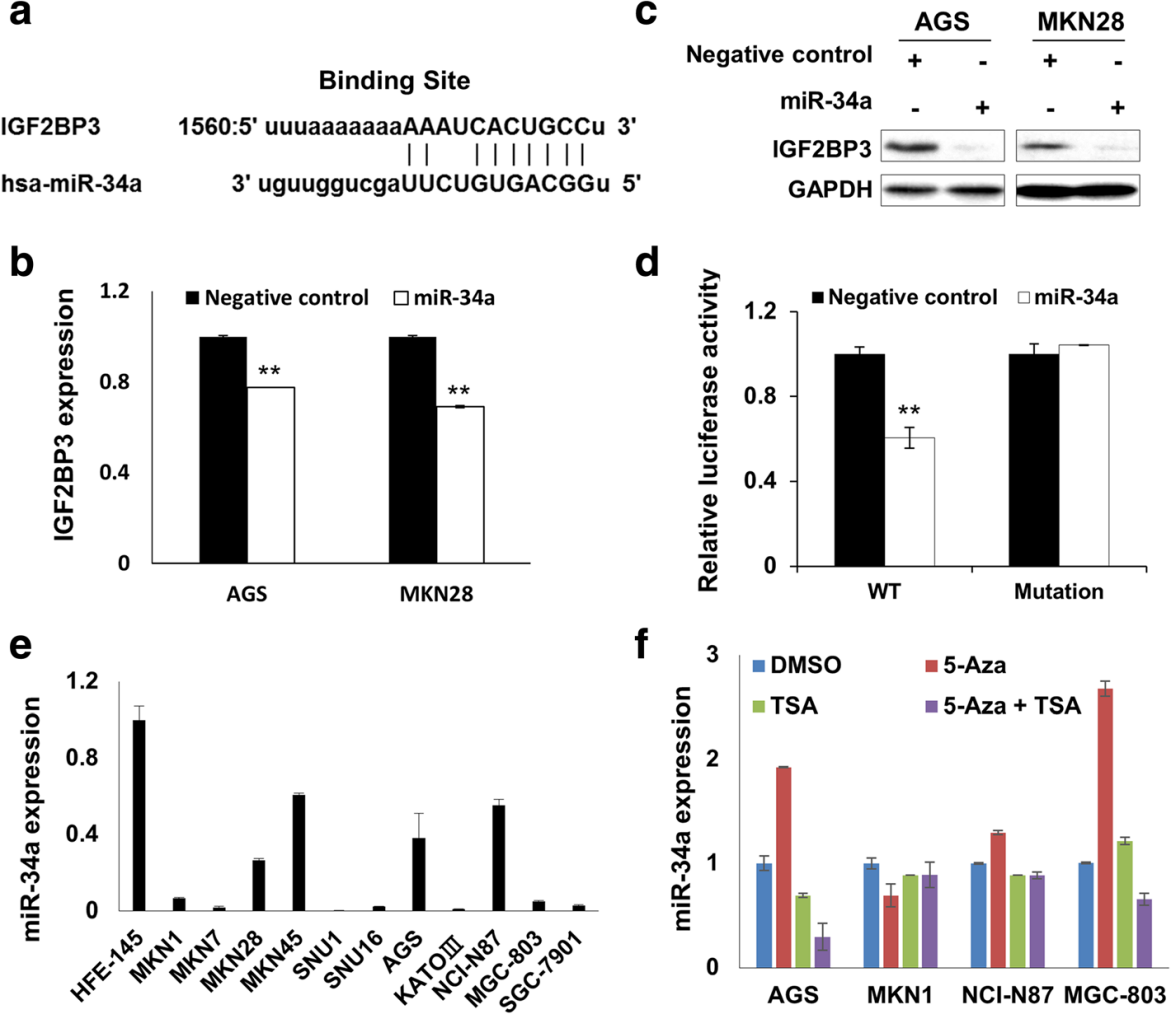
IGF2BP3 is a direct target of miR-34a in GC. **a** The putative miR-34a binding site in 3'UTR of IGF2BP3. **b** IGF2BP3 mRNA expression was down-regulated by ectopic miR-34a expression in AGS and MKN28 cells (**, *P* < 0.001). **c** miR-34a overexpression decreased the IGF2BP3 protein expression in GC cells.. **d** miR-34a inhibited the luciferase activity of constructs encompassing the wild type binding site, but the luciferase activity in the construct containing mutated binding site in IGF2BP3 3'UTR was not affected (WT, wild type of the complementary sequence for seed region; Mutation, the binding site was mutated; **, *P* < 0.001). **e** miR-34a expression in 11 GC cell lines compared with immortalized gastric epithelium cell HFE-145. **f** Expression of miR-34a in AGS, MKN1, NCI-N87 and MGC-803 cells after treatment with 5-Aza or TSA respectively

In IGF2BP3 3'UTR, the binding site for miR-34a was predicted by miRNA.org (http://www.microrna.org/) with the mirSVR scores of −1.098 (Fig. 4a). miR-34a ranks the top miRNA list that potentially targets IGF2BP3. Both the mRNA and protein expression of IGF2BP3 were found decreased in AGS and MKN28 cells after ectopic expression of miR-34a (Fig. 4b and c). To test whether IGF2BP3 was a direct target of miR-34a, we conducted the luciferase experiment. As shown in Fig. 4d, miR-34a inhibited the luciferase activity of construct encompassing the wild type binding site in IGF2BP3 3'UTR, but it had no effect on the construct containing mutated sequence of the binding site (Fig. 4d). This result supported that miR-34a regulated IGF2BP3 expression by directly binding with its 3'UTR. miR-34a showed uniformly down-regulated in all 11 GC cell lines compared with immortalized gastric epithelium cell line HFE-145 (Fig. 4e). However, the expression of miR-34a was not restored after treating the GC cells with 5-Aza or TSA (Fig. 4f), suggesting that epigenetic modification, such as promoter methylation or histone deacetylation, may not play an important role in the regulation of miR-34a expression.

### miR-34a functions as potential tumor suppressor in GC cells

**Fig. 5:**
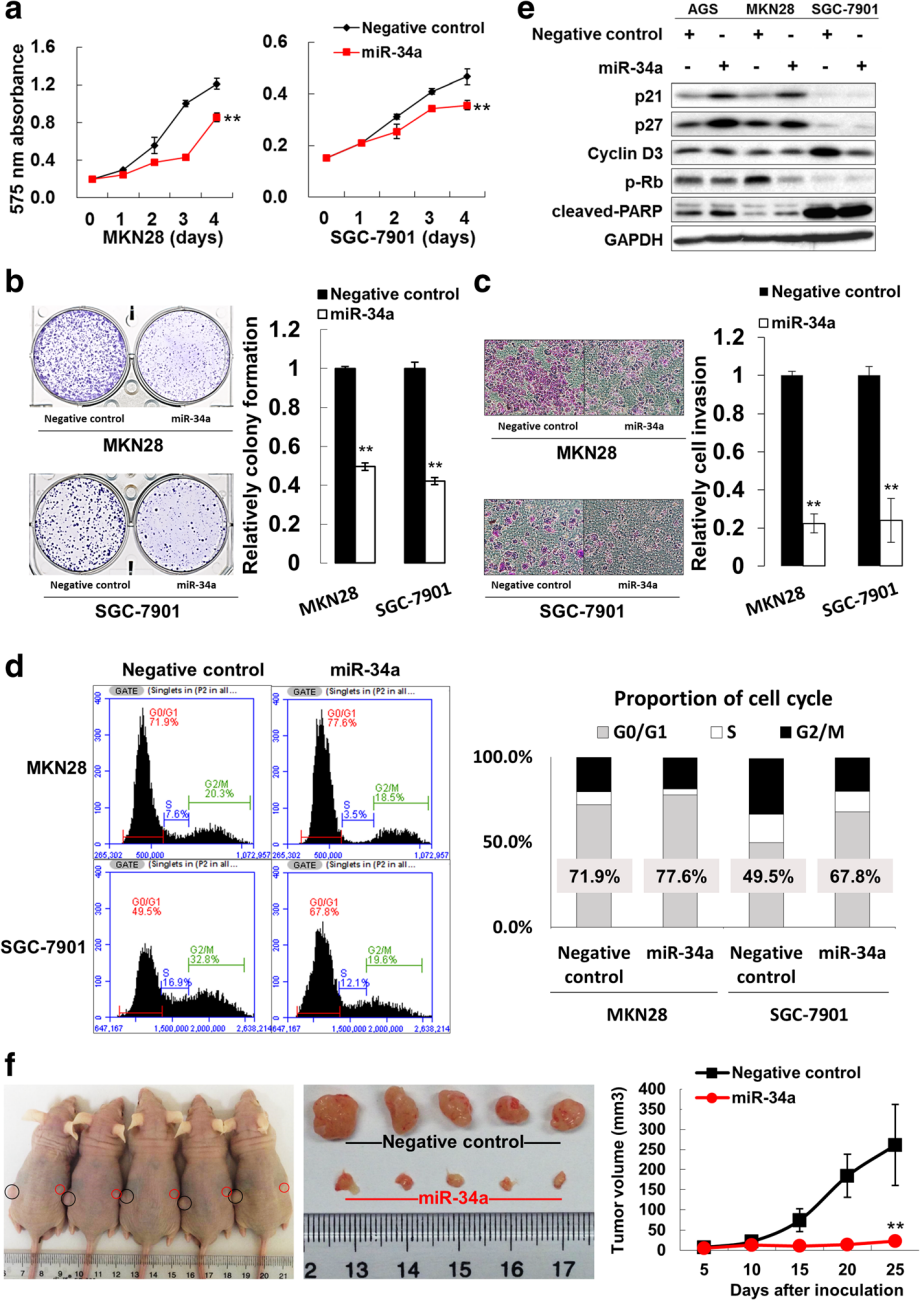
miR-34a is a tumor-suppressive miRNA. **a** Overexpressed miR-34a impaired GC cell growth in a 4-day MTT assay in MKN28 and SGC-7901 cells (**, *P* < 0.001). **b** Smaller sizes and less numbers of monolayer colonies were detected in miR-34a transfectants compared with negative control group (**, *P* < 0.001). **c** The cell invasion ability was partly abolished after treatment with miR-34a (**, *P* < 0.001). The invaded cells from the matrigel were counted in three random vision fields for getting standard deviations. **d** Cell cycle analysis by flow cytometry revealed that ectopic expression of miR-34a raised the proportion of G0/G1-phase cells. **e** Western blot analysis of related cell cycle regulators and apoptotic markers. **f** miR-34a overexpression in MGC-803 cells inhibited xenograft formation in vivo (**, *P* < 0.001)

To investigate the biological role of miR-34a in GC, ectopic expression of miR-34a precursor in GC cells (MKN28 and SGC-7901) was performed. MTT proliferation assays indicated that overexpression of miR-34a reduced cell proliferation (Fig. 5a) and this inhibitory effect was subsequently validated via monolayer colony formation study (Fig. 5b), with less and smaller colonies found in miR-34a over-expressed group. Moreover, a descending cell invasion was found in the cells with ectopic miR-34a transfection compared with negative control (Fig. 5c). Due to the proliferation inhibition, cell cycle analysis by flow cytometry was conducted. As shown in Fig. 5d, miR-34a increased G0/G1 proportion from 71.9 to 77.6% in MKN28 cells. In SGC-7901, miR-34a overexpression resulted in 67.8% proportion of G0/G1 cells when compared to 49.5% in negative control group (Fig. 5d). The results were further verified by Western blot of cell cycle regulators and apoptosis markers. The expression of p-Rb and Cyclin D3 showed decreased but p21 and p27 displayed up-regulated after overexpression of miR-34a in AGS, MKN28 and SGC-7901 cell. In addition, miR-34a induced late apoptosis, represented by the activation of cleaved-PARP in all the three GC cell lines (Fig. 5e). The effect of miR-34a on in vivo tumor growth was also investigated. MGC-803 cells transfected with negative control and miR-34a were subcutaneously injected into 4-week-old nude mice. Tumors grew slower and showed smaller size in miR-34a group than those in the negative control group (*P* < 0.001, Fig. 5f).

### Downregulation of miR-34a correlates with poor survival in GC

**Fig. 6:**
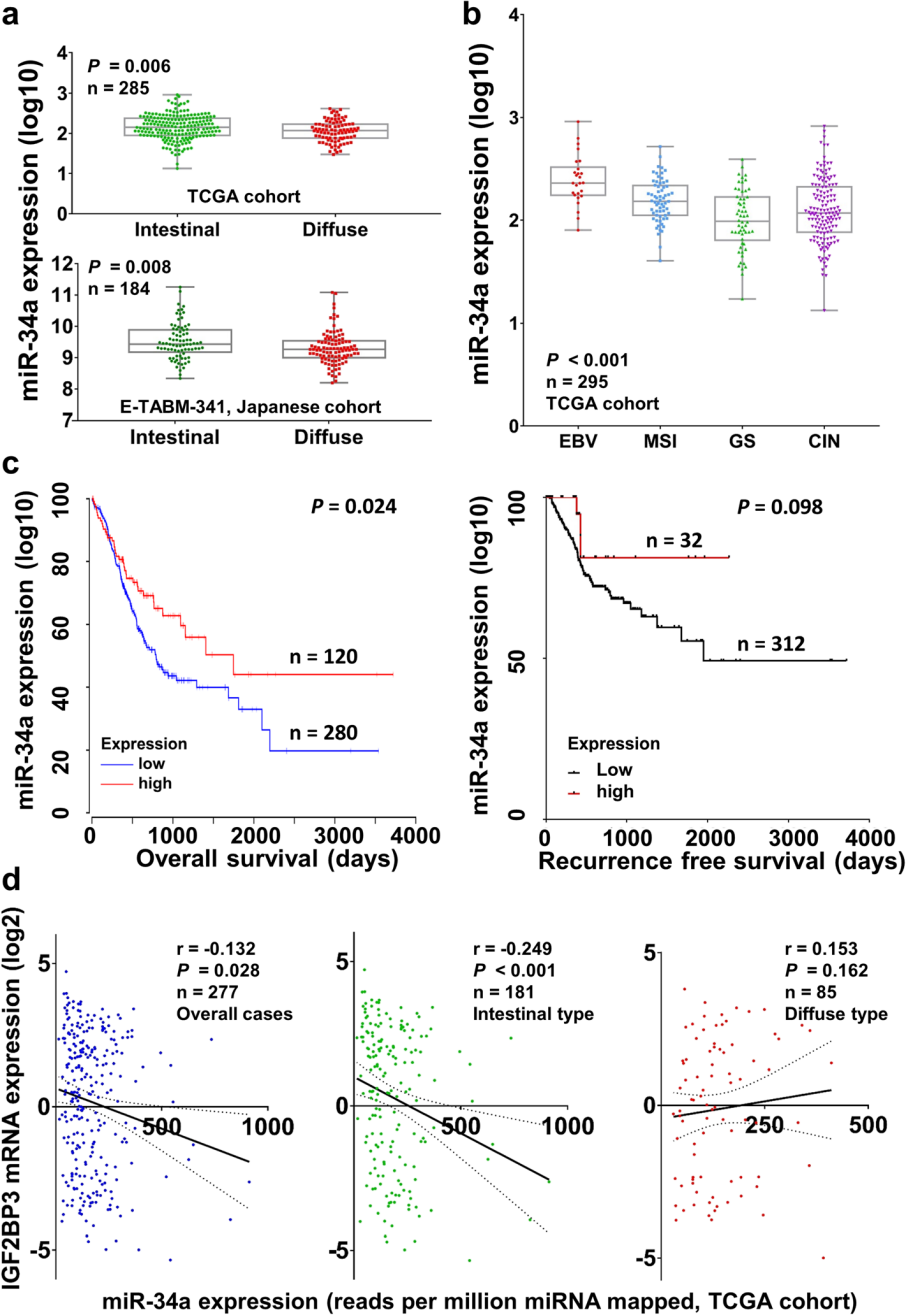
Low expression of miR-34a correlates with worse clinical outcome in GC patients. **a** A lower expression of IGF2BP3 was detected in primary diffuse type of GC compared with intestinal type GC in a published Japanese cohort (E-TABM-341, *n* = 184, *P* = 0.008) and TCGA cohort (*n* = 285, *P* = 0.008). **b** The expression of miR-34a in four molecular subtypes of GC (TCGA cohort, *n* = 295, *P* < 0.001). **c** In TCGA cohort, downregulation of miR-34a predicted a shorter overall survival in primary GC samples (*P* = 0.024). However for recurrence free survival, only an unfavorable trend was detected in low-expression miR-34a group (*P* = 0.098). **d** miR-34a expression showed inversely correlated with IGF2BP3 mRNA level in primary samples of TCGA cohort (*r* = −0.132, *n* = 277, *P* = 0.028). In intestinal type GC, the negative correlation was more stringent (*r* = −0.249, *n* = 181, *P* < 0.001)

Apart from the functional study, the expression of miR-34a in primary samples and its clinical correlation were analyzed. In two published cohorts, TCGA (*n* = 285) as well as a Japanese cohort (E-TABM-341, *n* = 184) [35], a considerably lower expression of IGF2BP3 was observed in diffuse type in contrast with intestinal type GC (*P* = 0.006 and *P* = 0.008 respectively, Fig. 6a). For molecular characterization of GC, miR-34a expression showed a lower expression in in GS subtype (*P* < 0.001, Fig. 6b), suggesting downregulation of miR-34a might strongly be associated with metastasis. Furthermore, downregulation of miR-34a in GC samples predicted poorer outcome in overall survival (*P* = 0.024) [36], but for recurrence free survival, decreased miR-34a only showed a trend to correlate with poor prognosis (*P* = 0.098, Fig. 6c and Additional file 7: Table S6). To further investigate if miR-34a is a crucial regulator of IGF2BP3 in GC, the expression correlation of miR-34a and IGF2BP3 was analyzed in TCGA cohort. As shown in Fig. 6d, there was a negative association between their expression in overall cases (*r* = −0.132, *n* = 277, *P* = 0.028). Remarkably, when the cohort was re-analyzed according to Lauren classification, a more stringent reverse correlation between miR-34a and IGF2BP3 was observed in intestinal type GC (*r* = −0.249, *P* < 0.001). These results implicated that IGF2BP3 was modulated by miR-34a in primary GC, especially in intestinal type GC.

### IGF2BP3 re-expression rescues the tumor-suppressive function of miR-34a

**Fig. 7:**
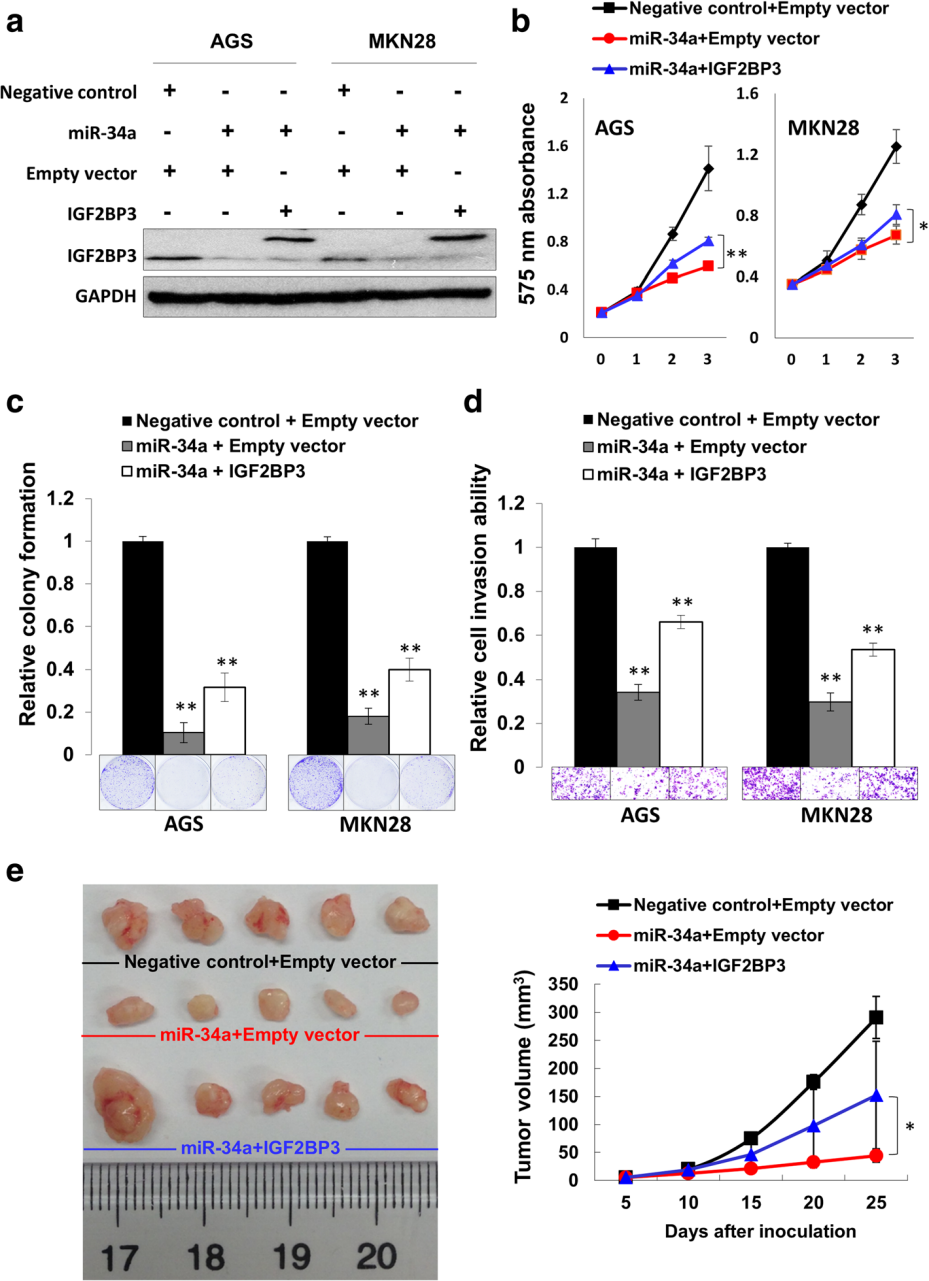
IGF2BP3 re-expression partly abrogated the inhibitory effect of miR-34a in GC. **a** Efficiency of IGF2BP3 re-expression was determined by Western blot. **b** MTT proliferation assay revealed that re-overexpressed IGF2BP3 partly rescued the growth repression of miR-34a (*, *P* < 0.05; **, *P* < 0.001). **c** Partial restoration of the suppressed cell growth was observed by monolayer colony formation after IGF2BP3 re-overexpression (**, *P* < 0.001). **d** Ectopic expression of IGF2BP3 partly recovered the cell invasion ability impaired by miR-34a (**, *P* < 0.001). **e** Re-expressed IGF2BP3 in MGC-803 cells partly revive the tumor volume compared with miR-34a group confirmed by in vivo study (*, *P* < 0.05)

Finally, we investigated if the IGF2BP3 was a real functional target of miR-34a in GC by rescue experiments. Re-expression efficiency of IGF2BP3 was first verified by Western blot analysis (Fig. 7a). Both MTT proliferation and colony formation assays explicated growth-inhibition effect of miR-34a was partly alleviated by IGF2BP3 re-overexpression (Fig. 7b and c). In addition, re-expression of IGF2BP3 partly restored the impaired cell invasion ability in miR-34a treated GC cells (Fig. 7d). Importantly, in vivo experiments demonstrated that re-expressed IGF2BP3 partly abolished the anti-carcinogenic function of miR-34a, further emphasizing that IGF2BP3 was the *bona fide* functional target of miR-34a in GC (Fig. 7e).

## Discussion

IGF2BP3, contains a structure of two N-terminal RNA recognition motifs (RRM) and four C-terminal messenger ribonucleoprotein K homology (KH) domains [37]. The C-terminal KH domains are required for RNA-binding, and this attribute decides the cytoplasmic localization and granular distribution of IGF2BP3 [38, 39].

IGF2BP3 has been reported to participate in tumorigenicity in numerous kinds of cancers and its overexpression in tumorous tissues makes it a promising biomarker for diagnosis or prognosis accordingly [40]. The enrichment of IGF2PB3 in human malignancies might promote tumor growth via raising the quantity of IGF2 [41]. Moreover, IGF2BP3 enhanced cell proliferation through synergizing with hnRNPM in the nucleus, leading to an elevated level of cyclins [42]. Recently, IGF2BP3 was believed to, mainly through the let-7 family, boost the expression level of High-mobility group AT-hook 2 (HMGA2) by preventing miRNA binding [43]. In breast cancer, apart from the capability of elevating the invasive potential [44], IGF2BP3 was also believed to be involved in chemo-resistance by stabilizing the mRNA of breast cancer resistance protein (ABCG2) [45].

There were several reports focusing on IGF2BP3 in GC in the past years. A group of Japanese scientists suggested IGF2BP3 to be an independent poor prognostic marker and an indicator for peritoneal dissemination in GC after surgery [46]. Similarly, merely based on their own clinical cohort, Kim HJ et al. also indicated that IGF2BP3 predicted worse outcome and malignant effusion among GC patients [13]. Recently, IGF2BP3 was again proposed to be associated with poor survival in Brazilian population [47]. However, all the previous studies failed to investigate the functional role of IGF2BP3 in gastric carcinogenesis. In our study, we provided the first evidence to comprehensively reveal the oncogenic function of IGF2BP3 in GC. Upregulation of IGF2BP3 was detected in both GC cell lines and primary GC samples in our cohort, as well as other published databases. In addition, we described IGF2BP3 expression with molecular classification proposed by TCGA. The IGF2BP3 was uniformly high expressed in all the four subtypes in contrast with normal gastric tissues. This abundance was proved to promote cell growth and invasive ability in GC cell lines via functional assays, which was further confirmed by GSEA results. Moreover, data from flow cytometry demonstrated that IGF2BP3 might play a role in G1 to M phase transition. And a previous work done by Rivera Vargas T et al. explained that IGF2BP3 protein directly bound with the mRNAs of CCND1/3, together with hnRNPM, to control the expression of CCND1/3 in the nucleus [42].

To further investigate the mechanism of IGF2BP3 upregulation in gastric carcinogenesis, we firstly checked the copy number change and mRNA expression in TCGA cohort. However, more cases with mRNA expression than the cases with copy number gain were detected, we thereby focused on post-transcriptional regulation mechanism, such as dysregulated related miRNAs. After screening and a series validation assays, miR-34a was confirmed to be the main regulator of IGF2BP3 and their expression showed negative correlation in primary samples, especially in intestinal type GC. Rescue experiments demonstrated that IGF2BP3 is a crucial target of miR-34a in GC because IGF2BP3 re-overexpression partly relieved the tumor-suppressive effect of miR-34a. miR-34a has been identified as a classical tumor suppressive miRNA in variety of malignancies [48, 49]. Multiple putative targets have been revealed for miR-34a to exert its tumor-suppressive role [50], such as period 1 (Per1) in cholangiocarcinoma [51], NOTCH1 in colon cancer stem cells [52], Hepatocyte nuclear factor 4α (HNF4α) in hepatocellular carcinoma [53] and transforming growth factor-β-induced factor 2 (Tgif2) in bone metastasis [54]. It has been also reported that miR-34a enhances the sensitivity of GC cells against cisplatin through PI3K/AKT/survivin pathway [50, 55]. On the other hand, our study suggested the activation of IGF2BP3 in GC is partly due to silence of miR-34a and enriched the target pool of miR-34a. Furthermore, a great number of papers have proposed that miR-34a mimics had therapeutic potential to inhibit cancer progression [56–58].

## Conclusions

In summary, high expression of IGF2BP3 in GC was associated with poor prognosis. Knockdown of IGF2BP3 significantly suppressed its oncogenic role. The upregulation of IGF2BP3 was partly due to the silence of tumor-suppressive miR-34a in some GC samples. Our findings not only clarified the mechanism of IGF2BP3 upregulation in GC but also provided therapeutic target for clinical intervention.

## Additional files

Additional file 1: Table S1. Oligonucleotides used in the luciferase activity experiments. The oligonucleotides were annealed and subcloned into pMIR-REPORT via *HindIII* and *SpeI* restriction sites. WT (Wild type), full length of the putative miRNA binding site; Mutation, the binding site was mutated. (DOC 34 kb)

Additional file 2: Table S2. Expression microarray. (XLS 4646 kb)

Additional file 3: Figure S1. The correlation of IGF2BP3 copy number changes with it mRNA expression in primary GC samples (*, *P* < 0.05; TCGA cohort). (TIF 1399 kb)

Additional file 4: Table S3. Statistical results of IGF2BP3 survival curve analyzed by KM Plotter (http://kmplot.com) (sig, significantly; CI, confident interval). (DOCX 14 kb)

Additional file 5: Table S4. Correlation of IGF2BP3 expression in GC with other clinicopathologic features (significant *P*-value in bold and Italic format). The case number and percentage counted were shown in the table. (DOC 64 kb)

Additional file 6: Table S5. Univariate and multivariate Cox regression analysis of the association between clinicopathologic characteristics and disease specific survival in patients with gastric adenocarcinoma (*n* = 247, significant *P*-value in bold and Italic format). (DOC 35 kb)

Additional file 7: Table S6. Statistical results of miR-34a survival curve derived from TCGA (sig, significantly; CI, confident interval). (DOCX 15 kb)

## Abbreviations

ABCG2: Breast cancer resistance protein
CIN: Chromosomal instability
GC: Gastric cancer
GS: Genomically stable
GSEA: Gene Set Enrichment Analysis
HMGA2: High-mobility group AT-hook 2.
HNF4α: Hepatocyte nuclear factor 4α
hnRNPM: Heterogeneous Nuclear Ribonucleoprotein M
IGF2BP3: Insulin-like growth factor-2 mRNA-binding protein 3
KH domains: K homology domains
miRNA: microRNAs
Per 1: Period 1
RRM: RNA recognition motifs
TCGA: The Cancer Genome Atlas
Tgif2: Transforming growth factor-β-induced factor 2
UTRs: Untranslated regions
WHO: World Health Organization
