# Supplementary material for: IGF2BP3 functions as a potential oncogene and is a crucial target of miR-34a in gastric carcinogenesis
Source: Mol Cancer. 2017 Apr 11;16:77. doi: 10.1186/s12943-017-0647-2 (PMC5387209; doi:10.1186/s12943-017-0647-2)
Supplement: Supplementary file 1 — Oligonucleotides used in the luciferase activity experiments. The oligonucleotides were annealed and subcloned into pMIR-REPORT via HindIII and SpeI restriction sites. WT (Wild type), full length of the putative miRNA binding site; Mutation, the binding site was mutated. (DOC 34 kb) [file 12943_2017_647_MOESM1_ESM.doc]

**Table S1** Oligonucleotides used in the luciferase activity experiments. The oligonucleotides were annealed and subcloned into pMIR-REPORT via *HindIII* and *SpeI* restriction sites. WT (Wild type), full length of the putative miRNA binding site; Mutation, the binding site was mutated.

|  | Oligonucleotides | Sequence (5’-3’) |
| --- | --- | --- |
| IGF2BP3 | miR-34a_WT_Sense | CTAGTTTTAAAAAAAAAATCACTGCCTA |
|  | miR-34a_WT_Antisense | AGCTTAGGCAGTGATTTTTTTTTTAAAA |
|  | miR-34a_Mutation_Sense | CTAGTTTTAAAAAAAAAATGTGACGGTA |
|  | miR-34a_Mutation_Sense | AGCTTACCGTCACATTTTTTTTTTAAAA |
|  |  |  |
