# Supplementary figures and images for: IGF2BP3 functions as a potential oncogene and is a crucial target of miR-34a in gastric carcinogenesis
Source: Mol Cancer. 2017 Apr 11;16:77. doi: 10.1186/s12943-017-0647-2 (PMC5387209; doi:10.1186/s12943-017-0647-2)

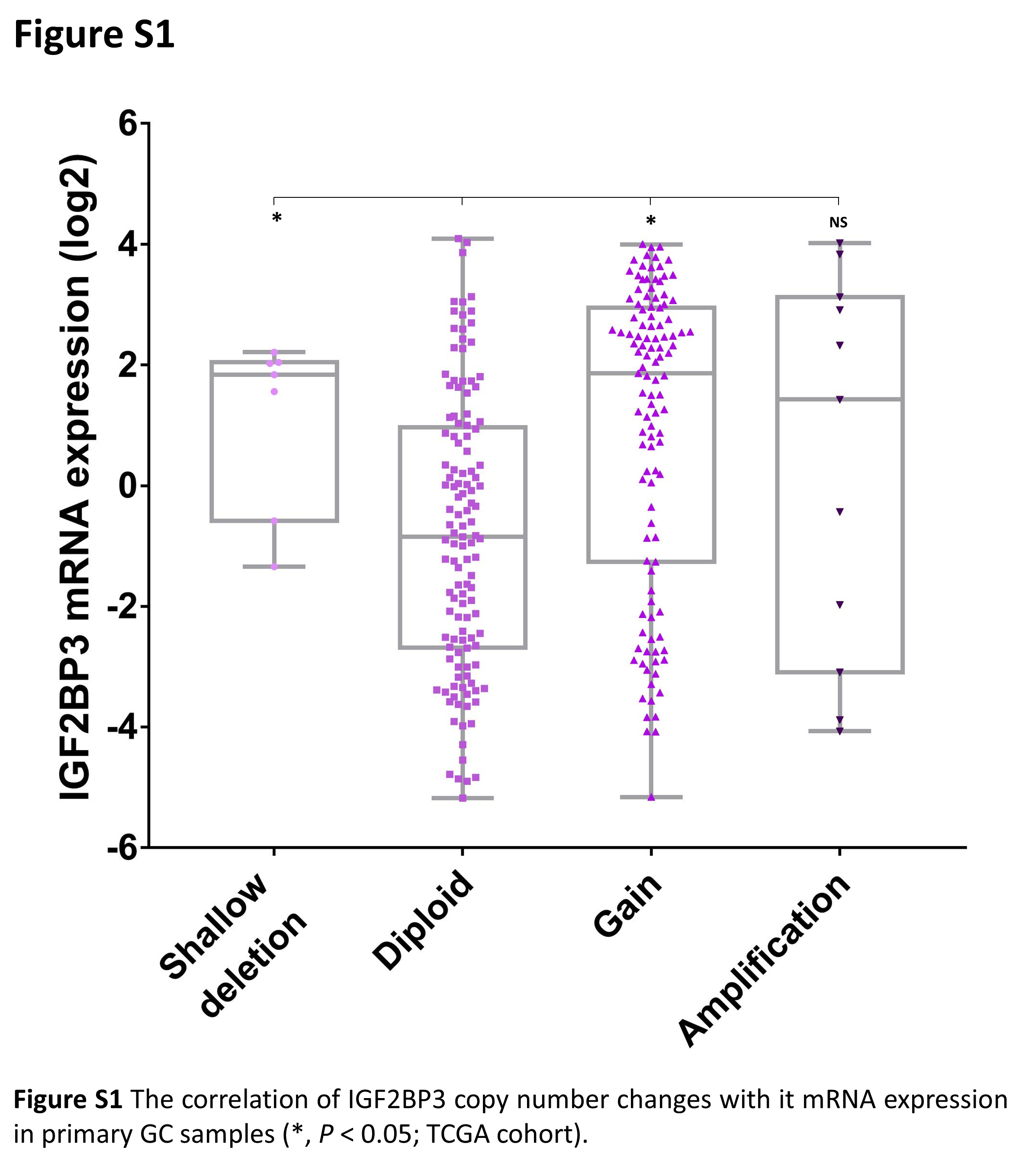

Supplement: Supplementary file 3 — The correlation of IGF2BP3 copy number changes with it mRNA expression in primary GC samples (*, P < 0.05; TCGA cohort). (TIF 1399 kb) [file 12943_2017_647_MOESM3_ESM.tif]
