# Supplementary material for: IGF2BP3 functions as a potential oncogene and is a crucial target of miR-34a in gastric carcinogenesis
Source: Mol Cancer. 2017 Apr 11;16:77. doi: 10.1186/s12943-017-0647-2 (PMC5387209; doi:10.1186/s12943-017-0647-2)
Supplement: Supplementary file 4 — Statistical results of IGF2BP3 survival curve analyzed by KM Plotter (http://kmplot.com) (sig, significantly; CI, confident interval). (DOCX 14 kb) [file 12943_2017_647_MOESM4_ESM.docx]

**Table S3** Statistical results of IGF2BP3 survival curve analyzed by KM Plotter (<http://kmplot.com>) (sig, significantly; CI, confident interval).

| Overall survival | | First progression survival | |
| --- | --- | --- | --- |
| Log-rank (Mantel-Cox) test | | Log-rank (Mantel-Cox) test | |
| Chi square | 5.572 | Chi square | 13.09 |
| Degree of freedom | 1 | Degree of freedom | 1 |
| *P*-value | 0.0182 | *P*-value | 0.0003 |
| Are the survival curves sig different? | Yes | Are the survival curves sig different? | Yes |
| Median survival | | Median survival | |
| IGF2BP3 high | 23.6 | IGF2BP3 high | 12.6 |
| IGF2BP3 low | 32.6 | IGF2BP3 low | 25.2 |
| Ratio | 0.7239 | Ratio | 0.5 |
| 95% CI of ratio | 0.6095 - 0.8599 | 95% CI of ratio | 0.4092 - 0.611 |
| Hazard Ratio (logrank) | high/low | Hazard Ratio (logrank) | high/low |
| Ratio | 1.229 | Ratio | 1.442 |
| 95% CI of ratio | 1.031 - 1.466 | 95% CI of ratio | 1.173 - 1.773 |
