# Supplementary material for: IGF2BP3 functions as a potential oncogene and is a crucial target of miR-34a in gastric carcinogenesis
Source: Mol Cancer. 2017 Apr 11;16:77. doi: 10.1186/s12943-017-0647-2 (PMC5387209; doi:10.1186/s12943-017-0647-2)
Supplement: Supplementary file 5 — Correlation of IGF2BP3 expression in GC with other clinicopathologic features (significant P-value in bold and Italic format). The case number and percentage counted were shown in the table. (DOC 64 kb) [file 12943_2017_647_MOESM5_ESM.doc]

**Table S4** Correlation of IGF2BP3 expression in GC with other clinicopathologic features (significant *P*-value in bold and Italic format). The case number and percentage counted were shown in the table.

|  |  | | Gastric cancer (n = 247) | | |
| --- | --- | --- | --- | --- | --- |
|  |  | Negative/Weak (%) | | Moderate/Strong (%) | *P*-value |
| Sex | M | | 63 (38.2) | 102 (61.8) | 0.379 |
|  | F | | 26 (31.7) | 56 (68.3) |  |
| Age | <=60 | | 32 (35.2) | 59 (64.8) | 0.303 |
|  | >60 | | 57 (36.5) | 99 (63.5) |  |
| Type | Intestinal | | 58 (43.0) | 77 (57.0) | 0.237 |
|  | Diffuse | | 31 (27.9) | 80 (72.1) |  |
| Grade | 1 | | 3 (37.5) | 5 (62.5) | 0.513 |
|  | 2 | | 38 (42.7) | 51 (57.3) |  |
|  | 3 | | 47 (32.0) | 100 (68.0) |  |
| Stage | 1 | | 18 (33.3) | 36 (66.7) | 0.194 |
|  | 2 | | 14 (50.0) | 14 (50.0) |  |
|  | 3 | | 36 (45.0) | 44 (55.0) |  |
|  | 4 | | 20 (24.4) | 62 (75.6) |  |
| Stage (T) | 1 | | 9 (26.5) | 25 (73.5) | 0.421 |
|  | 2 | | 34 (48.6) | 36 (51.4) |  |
|  | 3 | | 39 (31.0) | 87 (69.0) |  |
|  | 4 | | 5 (41.7) | 7 (58.3) |  |
| Stage (N) | 0 | | 20 (37.7) | 33 (62.3) | ***0.025*** |
|  | 1 | | 28 (41.8) | 39 (58.2) |  |
|  | 2 | | 30 (41.1) | 43 (58.9) |  |
|  | 3 | | 10 (19.6) | 41 (80.4) |  |
| Stage (M) | 0 | | 81 (38.9) | 127 (61.1) | 0.636 |
|  | 1 | | 8 (21.1) | 30 (78.9) |  |
| Lymph Node | 0 | | 20 (37.7) | 33 (62.3) | 0.590 |
|  | 1 | | 68 (35.4) | 124 (64.6) |  |
| *H. pylori* | Absence | | 37 (33.6) | 73 (66.4) | 0.109 |
|  | Presence | | 48 (39.3) | 74 (60.7) |  |
