# Supplementary material for: IGF2BP3 functions as a potential oncogene and is a crucial target of miR-34a in gastric carcinogenesis
Source: Mol Cancer. 2017 Apr 11;16:77. doi: 10.1186/s12943-017-0647-2 (PMC5387209; doi:10.1186/s12943-017-0647-2)
Supplement: Supplementary file 6 — Univariate and multivariate Cox regression analysis of the association between clinicopathologic characteristics and disease specific survival in patients with gastric adenocarcinoma (n = 247, significant P-value in bold and Italic format). (DOC 35 kb) [file 12943_2017_647_MOESM6_ESM.doc]

**Table S5** Univariate and multivariate Cox regression analysis of the association between clinicopathologic characteristics and disease specific survival in patients with gastric adenocarcinoma (n = 247, significant P-value in bold and Italic format).

|  | Univariate analysis | Multivariate analysis |
| --- | --- | --- |
| Sex | 0.224 |  |
| Age | ***0.027*** | ***< 0.001*** |
| Type | ***< 0.001*** | 0.678 |
| Grade | ***0.008*** | 0.627 |
| Stage | ***< 0.001*** | ***< 0.001*** |
| Lymph Node | ***< 0.001*** | 0.880 |
| *H. pylori* | 0.269 |  |
| IGF2BP3 | ***0.013*** | 0.063 |
