# Supplementary material for: IGF2BP3 functions as a potential oncogene and is a crucial target of miR-34a in gastric carcinogenesis
Source: Mol Cancer. 2017 Apr 11;16:77. doi: 10.1186/s12943-017-0647-2 (PMC5387209; doi:10.1186/s12943-017-0647-2)
Supplement: Supplementary file 7 — Statistical results of miR-34a survival curve derived from TCGA (sig, significantly; CI, confident interval). (DOCX 15 kb) [file 12943_2017_647_MOESM7_ESM.docx]

**Table S6** Statistical results of miR-34a survival curve derived from TCGA (sig, significantly; CI, confident interval).

| Overall survival | |  | First progression survival | |
| --- | --- | --- | --- | --- |
| Log-rank (Mantel-Cox) test | | | Log-rank (Mantel-Cox) test | |
| Chi square | 6.893 |  | Chi square | 2.731 |
| Degree of freedom | 1 |  | Degree of freedom | 1 |
| *P*-value | 0.0087 |  | *P*-value | 0.0984 |
| Are the survival curves sig different? | Yes |  | Are the survival curves sig different? | No |
| Median survival | |  | Median survival | |
| miR-34a high | 1686 |  | miR-34a high | undefined |
| miR-34a low | 801 |  | miR-34a low | 1949 |
| Ratio | 2.105 |  | Ratio | - |
| 95% CI of ratio | 1.441 - 3.074 | | 95% CI of ratio | - |
| Hazard Ratio (logrank) | high/low |  | Hazard Ratio (logrank) | high/low |
| Ratio | 0.6057 |  | Ratio | 0.3923 |
| 95% CI of ratio | 0.433 - 0.8472 | | 95% CI of ratio | 0.1821 - 0.845 |
